# The association between perceived usefulness of AI-generated art tools and self-reported creative design performance: a parallel mediation model of artistic perception, cognitive engagement, and aesthetic judgment

**DOI:** 10.3389/fpsyg.2026.1755561

**Published:** 2026-06-11

**Authors:** Zhang Guosheng, Meng Lei

**Affiliations:** Art College, Qilu Normal University, Jinan, China

**Keywords:** aesthetic judgment, artistic perception, cognitive engagement, mediation model, perceived usefulness of AI-generated art tools, self-reported creative design performance

## Abstract

**Introduction:**

The rapid expansion of AI-generated art tools has transformed creative production, yet its psychological mechanisms in shaping design outcomes remain underexplored. This study examines the associations between perceived usefulness of AI-generated art tools and self-reported creative design performance through three parallel mediating pathways: artistic perception, cognitive engagement, and aesthetic judgment.

**Methods:**

Guided by aesthetic processing and cognitive engagement theories, a questionnaire survey was administered to Chinese university students enrolled in design-related programs, generating 387 valid responses. Data were analyzed using partial least squares structural equation modeling.

**Results:**

The results show that perceived usefulness of AI-generated art tools is positively associated with all three mediators, each of which is positively associated with self-reported creative design outcomes. Cognitive engagement emerged as the strongest mediator, followed by aesthetic judgment and artistic perception. A significant direct effect also remained, indicating partial mediation. The overall model explained 53.2 percent of the variance in self-reported creative design performance, demonstrating both strong explanatory power and meaningful predictive relevance.

**Discussion:**

These findings highlight the multifaceted ways in which perceived usefulness of AI-generated art tools is associated with the perceptual, cognitive, and evaluative processes that underpin creative design, offering valuable implications for design education, digital creativity research, and the integration of AI tools in creative industries.

## Introduction

The rapid evolution of artificial intelligence has brought profound shifts to creative domains, particularly in visual design (Almogren, 2025; Zhang, 2024), where generative models now produce high-quality artistic outputs that rival human-made work. These AI-generated images increasingly appear in classrooms, design studios, and creative industries (Amanbay, 2023), influencing how individuals conceptualize visual aesthetics and develop creative ideas. As AI becomes more embedded in creative processes, understanding how humans psychologically respond to AI-generated art has become an important question for scholars and practitioners alike (Hitsuwari et al., 2023). Yet, empirical research exploring this relationship remains limited despite the widespread adoption of AI tools in design education and professional practice.

The incorporation of AI-generated stimuli into creative workflows provides learners and designers with access to diverse artistic patterns, unconventional visual combinations, and novel aesthetic cues. These characteristics may be associated with richer visual imagination, greater sensitivity to artistic features, and deeper forms of cognitive processing. Early studies highlight how generative tools can promote ideational variety and stimulate creative thinking (Daju and Moldovan, 2024; Koivisto and Grassini, 2023). However, most existing work treats AI either as a technological artifact or as a tool for generating creative outputs, rather than as a stimulus capable of influencing the perceptual and cognitive mechanisms that underpin creative performance. This theoretical oversight suggests the need to explore how individuals psychologically engage with AI-generated art and how such engagement supports creative design outcomes.

Creativity scholars emphasize that strong creative performance does not emerge solely from technical skills or idea generation. Instead, creative work is deeply shaped by how individuals perceive visual cues, cognitively process those cues, and evaluate aesthetic qualities (Agustini et al., 2022; Szostak, 2023). These internal mechanisms collectively determine how visual information is transformed into original and meaningful design solutions (Tigre Moura et al., 2023). While this perspective is well established, limited research examines how contemporary AI-generated art activates these psychological processes. Traditional studies have focused on responses to human-made art, natural scenes, or designer prototypes, leaving unanswered questions about how AI-generated imagery—with its distinctive patterns, unexpected compositions, and algorithmic aesthetics—shapes the perceptual and cognitive foundations of creativity.

A clear theoretical gap emerges from this omission. Existing frameworks rarely consider AI-generated art as an input that might simultaneously influence perceptual awareness, deepen cognitive engagement, and heighten aesthetic judgment. These constructs are often examined independently or in different research traditions (e.g., Cheng, 2022; Hansdottir et al., 2022; Samo and Highhouse, 2023), resulting in fragmented insights. As generative AI becomes increasingly intertwined with learning and creative practice, there is a pressing need to understand how these psychological components interact when individuals engage with AI-generated artistic content. Addressing this gap is crucial not only for advancing creativity theory but also for informing design education, where AI tools are rapidly becoming part of everyday practice.

The present study addresses this theoretical gap by proposing a parallel mediation model that examines associations between perceived usefulness of AI-generated art tools and self-reported creative design performance through interconnected perceptual, cognitive, and aesthetic pathways. By examining how learners respond to AI-produced artistic stimuli, this research advances conceptual understanding of AI-assisted creativity and highlights the psychological mechanisms that explain how perceived usefulness of generative art tools is associated with self-reported creative design outcomes. The findings have significant implications for design pedagogy, digital creativity research, and the broader integration of AI into creative industries, offering a clearer understanding of how human creativity can be supported rather than overshadowed by artificial intelligence.

### Theory and hypotheses

Advances in generative artificial intelligence have renewed scholarly attention toward how individuals perceptually and cognitively interact with novel forms of visual stimuli. Theories of aesthetic processing suggest that meaningful engagement with visual information involves sequential stages, beginning with perceptual attention to artistic features, followed by deeper cognitive interpretation and evaluative judgment that together influence creative output (Pelowski et al., 2017). These frameworks imply that creative performance does not arise solely from technical proficiency but is shaped by how individuals make sense of the aesthetic complexity embedded in visual materials. AI-generated art, characterized by vivid textures, unexpected stylistic combinations, and algorithmic abstractions, provides particularly rich stimuli for activating such processes. Understanding these mechanisms is essential for clarifying how exposure to AI-generated imagery is associated with self-reported creative design performance.

Research on creativity consistently highlights the role of internal psychological processes in transforming external stimuli into innovative outcomes. The componential theory of creativity emphasizes that perceptual sensitivity, cognitive involvement, and aesthetic evaluation jointly influence the originality and usefulness of creative work (Amabile and Pratt, 2016). Similarly, dual-process perspectives on design cognition suggest that exposure to complex visual cues engages both intuitive and analytical processing, fueling idea generation and refinement (Evans, 2011). Although these theories offer strong foundations, they have been applied primarily to human-created artwork, natural scenes, or conventional design references. Much less is known about how AI-generated art—which often includes stylistic exaggerations, algorithmic distortions, and novel recombinations—activates these perceptual and cognitive pathways.

**Figure 1:**
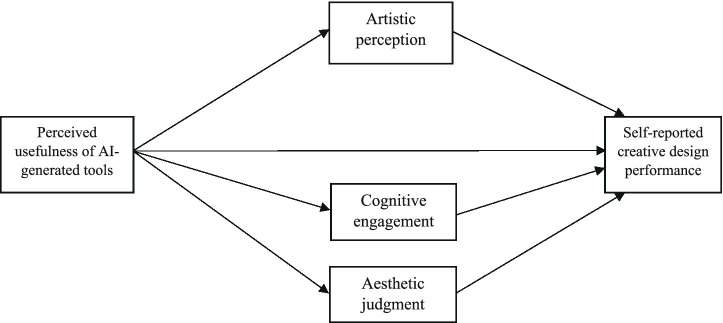
Conceptual model.

The current study integrates these theoretical perspectives… to propose that perceived usefulness of AI-generated art tools is associated with self-reported creative design performance through three interconnected mechanisms: heightened artistic perception, deeper cognitive engagement, and more refined aesthetic judgment. This approach expands existing theory by treating AI-generated art not as a tool or output but as a psychological stimulus associated with how learners interpret, process, and appraise visual information. The following hypotheses articulate these pathways in detail (Figure 1).

### Justification for parallel mediation model

Although aesthetic-processing theories often describe perceptual, cognitive, and evaluative responses as interconnected processes, prior creativity and aesthetic research suggests that these mechanisms may operate simultaneously rather than as strictly fixed sequential stages (Amabile and Pratt, 2016; Pelowski et al., 2017). Studies in creativity and design cognition further indicate that perceptual sensitivity, cognitive elaboration, and evaluative judgment can independently contribute to creative engagement when individuals interact with complex and visually novel stimuli (Ansorge et al., 2022; Tigre Moura et al., 2023). In the context of AI-generated art, learners may concurrently experience heightened perceptual attention, deeper cognitive involvement, and evaluative reflection while engaging with visually diverse and algorithmically generated imagery. Therefore, the present study conceptualizes artistic perception, cognitive engagement, and aesthetic judgment as parallel mediators to capture their distinct yet complementary explanatory roles in the association between perceived usefulness of AI-generated art tools and self-reported creative design performance.

### Perceived usefulness of AI-generated art tools and self-reported creative design performance

The expanding presence of AI-generated art in creative environments has introduced new forms of visual stimuli that designers frequently encounter during ideation and exploration. Prior research in creativity and design psychology shows that exposure to diverse, high-variation visual references support creative performance because it widens the range of cues available for reinterpretation and combination (Auernhammer and Roth, 2021; Zhang, 2024). AI-generated artworks often contain unconventional textures, color dynamics, and stylistic hybrids that differ markedly from traditional design references (Magni et al., 2024). These characteristics provide learners with stimuli that are inherently rich, surprising, and visually complex, thus offering more opportunities for reinterpretation during design work.

Studies focusing on digital creativity further suggest that generative AI can expand designers’ ideation boundaries by offering rapid access to varied aesthetic possibilities and alternative design directions (Chandrasekera et al., 2025). When individuals interact with such outputs, they are exposed to stylistic irregularities, unexpected patterns, and visually provocative combinations that can stimulate novel thinking. This aligns with long-standing creativity research showing that external stimuli influence creative performance when they offer cues that disrupt habitual patterns and prompt alternative associations (Albakry et al., 2025). Accordingly, AI-generated art functions not only as a tool but also as a source of inspiration that may be associated with greater originality and visual diversity in creative outcomes.

Bringing these perspectives together, a direct positive association can be expected between perceived usefulness of AI-generated art tools and self-reported creative design performance. The richness and variability inherent in AI-generated imagery provide designers with new aesthetic triggers, more options for visual exploration, and greater flexibility in forming original design concepts. Even before accounting for deeper perceptual or cognitive mechanisms, exposure to these stimuli may correspond with higher perceived quality and novelty in creative design outputs.

*H1*: Perceived usefulness of AI-generated art tools is positively associated with self-reported creative design performance.

### Mediating role of artistic perception

Visual materials that contain unfamiliar structures, high novelty, or stylistic disruption tend to draw intensified perceptual attention, encouraging individuals to scrutinize visual elements more closely. Research in art perception shows that exposure to complex or ambiguous images increases viewers’ focus on form, texture, contrast, and compositional relationships as they attempt to decode unfamiliar patterns (Szostak, 2023). Studies in visual design similarly report that atypical or surprising stimuli sharpen perceptual awareness because designers actively search for meaningful structures and aesthetic cues (Guerra-Tamez, 2023; Oksanen et al., 2023). AI-generated art, known for its unusual stylistic blends and algorithmically produced motifs, falls directly into this category. Empirical work indicates that generative outputs often include exaggerated details, unexpected combinations, and hybridized styles that prompt viewers to engage more attentively with visual information (Ansorge et al., 2022; Valencia et al., 2024). Such features increase the likelihood that individuals will notice subtle artistic elements and develop stronger perceptual sensitivity over time.

Creativity scholarship further demonstrates that perceptual responsiveness is tightly linked with creative performance. Designers who exhibit higher aesthetic sensitivity tend to identify visual opportunities more effectively, extract richer cues from artistic references, and integrate these cues into more original design outcomes (Bahrami-Ehsan et al., 2015). Prior research in digital and AI-assisted creativity also shows that repeated exposure to varied and visually complex stimuli enhances individuals’ ability to discern stylistic differences and recognize meaningful patterns, ultimately strengthening creative ideation (Jongmans et al., 2022; Tigre Moura et al., 2023). When considered together, these findings suggest that AI-generated art—because of its inherent novelty, diversity, and visual richness—serves as a particularly potent stimulus for developing perceptual refinement in design learners. Individuals who engage with such imagery are likely to become more attuned to artistic features, enhancing their capacity to process and reinterpret visual information.

Building on this evidence, it is reasonable to expect that perceived usefulness of AI-generated art tools is positively associated with artistic perception.

*H2*: Perceived usefulness of AI-generated art tools is positively associated with artistic perception.

The mediating role of artistic perception arises from its foundational influence on creative outcomes. When individuals develop stronger perceptual sensitivity, they are better equipped to decode aesthetic structures, identify promising visual directions, and repurpose fine-grained artistic cues in their own design work. Empirical studies show that designers with heightened perceptual awareness produce more coherent, aesthetically compelling, and novel outputs because they draw inspiration from a broader and more detailed visual knowledge base (Anderson et al., 2022; Ansorge et al., 2022). AI-generated art enhances this process by exposing learners to imagery that demands close inspection (Wohl, 2021), thereby enriching the pool of aesthetic insights available during creative production. The enhanced perceptual responsiveness triggered by AI-generated stimuli is therefore expected to strengthen the link between AI-based visual exposure and creative design outcomes.

*H3*: Artistic perception mediates the relationship between perceived usefulness of AI-generated art tools and self-reported creative design performance.

### Mediating role of cognitive engagement

Creative work often requires sustained mental effort, deep concentration, and active cognitive involvement, especially when individuals encounter stimuli that are novel, complex, or difficult to interpret. Prior research consistently shows that exposure to visually rich materials increases cognitive elaboration because individuals attempt to make sense of unfamiliar patterns, analyze structural elements, and explore potential meanings embedded in the imagery (Hansdottir et al., 2022; Shi et al., 2023). AI-generated art frequently contains unexpected stylistic blends, intricate details, and algorithmic variations that differ from traditional design references (Amanbay, 2023). These characteristics naturally demand higher levels of mental processing, prompting learners to think more deeply, compare alternatives, and evaluate multiple interpretations of the visual content. Studies in digital creativity similarly report that generative AI tools stimulate cognitive activation by presenting outputs that challenge conventional visual expectations, thereby encouraging more intensive reflection and idea elaboration (Chandrasekera et al., 2025; Daju and Moldovan, 2024).

Existing research in design cognition further emphasizes that creative tasks are strengthened when individuals engage in deeper cognitive processing. Designers who invest greater cognitive effort—through analyzing possibilities, refining concepts, and questioning assumptions—tend to generate more innovative and sophisticated outcomes (Caliskan and Altun, 2025). Cognitive engagement, defined as a state of focused mental involvement in a task, has been shown to facilitate meaningful learning and enhance problem-solving quality in creative contexts (Tantawy et al., 2021). When learners interact with stimuli that provoke curiosity, surprise, or cognitive challenge, they become more mentally absorbed, which in turn supports sustained exploration and more thoughtful design development.

Given this evidence, perceived usefulness of AI-generated art tools may be associated with higher cognitive engagement because its visual novelty and unpredictability stimulate interest, analytical thinking, and deeper mental absorption. Learners who regularly interact with such complex imagery may therefore become more cognitively invested in their creative tasks.

*H4*: Perceived usefulness of AI-generated art tools is positively associated with cognitive engagement.

The mediating role of cognitive engagement becomes apparent when considering its documented link to creative performance. Individuals who are more cognitively immersed tend to explore a wider range of design possibilities, engage in more detailed refinement of ideas, and integrate external cues more effectively into their creative processes (Agustini et al., 2022). Studies in digital and AI-assisted creativity further show that cognitive involvement enhances learners’ ability to transform visual stimuli—including those produced by AI—into coherent and original outcomes (Guan et al., 2023; Tang and Hew, 2022). When AI-generated art triggers deeper cognitive engagement, it provides the motivational and cognitive conditions necessary for high-quality design output. This suggests that cognitive engagement may help explain the association between exposure to AI-generated imagery and self-reported creative design performance.

*H5*: Cognitive engagement mediates the relationship between perceived usefulness of AI-generated art tools and self-reported creative design performance.

### Mediating role of aesthetic judgment

Aesthetic evaluation forms an essential component of how individuals interpret and respond to visual material, especially in creative disciplines where decisions about balance, coherence, harmony, and expressive quality shape the final outcome. Prior research shows that when individuals encounter visually complex or unconventional imagery, they engage in deeper aesthetic appraisal to determine meaning, emotional resonance, stylistic fit, and overall artistic value (Di Dio et al., 2025; Samo and Highhouse, 2023). AI-generated art often includes unexpected visual combinations, hybrid styles, and algorithmic distortions that challenge viewers’ expectations, making evaluative interpretation more active and deliberate (Bazin and Korica, 2021). Empirical findings in digital art contexts similarly show that generative outputs prompt viewers to reflect on aesthetic structure and emotional tone more intensely than standard reference images, as they must reconcile unfamiliar visual patterns with their internal aesthetic standards (Juslin et al., 2023; Yeh et al., 2021).

Research in creativity and design also emphasizes that aesthetic judgment plays a central role in determining the quality of creative output. Designers rely on evaluative reasoning to select appropriate ideas, refine stylistic directions, and ensure that their work maintains both originality and aesthetic coherence (Zhao et al., 2023). Stronger aesthetic judgment has been associated with higher-quality visual decisions because individuals with more refined evaluative skills are better able to assess the expressive and functional strengths of design elements (Albakry et al., 2025; Anderson et al., 2022; Cheng, 2022). In AI-assisted creativity, recent studies suggest that interacting with generative imagery encourages individuals to engage in comparative evaluation—judging what works, what does not, and which elements can be integrated into new ideas (Di Dio et al., 2025). This reflective evaluation strengthens their capacity to critically assess visual material and align it with creative intentions.

Drawing from these insights, perceived usefulness of AI-generated art tools may be associated with aesthetic judgment because its novelty and stylistic complexity encourage viewers to evaluate visual stimuli more thoroughly, consider alternative interpretations, and make more purposeful aesthetic decisions.

*H6*: Perceived usefulness of AI-generated art tools is positively associated with aesthetic judgment.

The mediating role of aesthetic judgment can be understood by examining how evaluative processes inform creative decisions. When individuals develop stronger aesthetic reasoning, they are more capable of discerning which visual elements should be adopted, adapted, or discarded in the pursuit of creative goals. Research shows that individuals who possess refined aesthetic judgment produce more coherent, emotionally compelling, and visually meaningful creative outcomes because they are able to integrate aesthetic insights into each stage of the design process (Chaudhuri et al., 2024; Juslin et al., 2023). AI-generated art facilitates this process by exposing learners to a diverse array of stylistic possibilities, prompting them to reflect on the aesthetic qualities of the imagery and make more deliberate judgments. This enrichment of evaluative capacity strengthens the connection between exposure to AI-generated stimuli and creative design performance.

*H7*: Aesthetic judgment mediates the relationship between perceived usefulness of AI-generated art tools and self-reported creative design performance.

## Methodology

Data for this study were collected from Chinese respondents because China represents one of the world’s fastest-growing creative-technology ecosystems, where AI-generated art tools are widely adopted in both academic and professional design settings (Mishkin-Drezner, 2024; Zhao et al., 2023). This context offers a theoretically meaningful platform to examine how exposure to AI-generated artistic content is associated with cognitive and aesthetic processes in real design-related tasks. The target population consisted of university students enrolled in design, media, communication, and digital-art programs, as these groups regularly interact with AI-assisted creative tools and therefore provide reliable insights for the study’s conceptual model.

A cluster-based convenience sampling approach was used to recruit participants from three large universities located in Fujian and Guangdong provinces. The questionnaires were distributed across three public universities located in Fujian and Guangdong provinces that offer design, media, and digital-art programs. Approximately 150 questionnaires were distributed at each institution to ensure balanced representation across the participating universities. Further, the questionnaires were distributed in person to students enrolled in design-related programs across different year levels. A total of 450 paper-based questionnaires were distributed, of which 401 were returned. After excluding responses containing substantial missing data, invariant response patterns, and obvious straight-lining behavior across scale items, 387 valid questionnaires were retained for analysis, resulting in an effective response rate of 86%. The raw response rate was 89.1% (401/450), while the final usable response rate after data screening was 86.0% (387/450). The sample size exceeded the minimum requirement suggested for PLS-SEM, which recommends at least 10 times the maximum number of indicators associated with a single construct, thereby ensuring adequate statistical power and model stability (Hair et al., 2019; Kock, 2018). Each questionnaire packet included a cover letter explaining the purpose of the study, assurances of confidentiality, voluntary participation, and instructions for completing the survey.

The final sample included approximately 52% female and 48% male respondents. In terms of age, around 61% were between 18 and 22 years old, 27% were between 23 and 25 years old, and the remaining 12% were above 25. Academic majors were distributed across design and visual arts (46%), media and communication (32%), computer-assisted creative technologies (14%), and other related disciplines (8%). These demographic patterns provide a useful context for examining associations between AI-generated art and self-reported creative design performance among Chinese creative-arts learners, although the findings should not be generalized beyond similar educational settings without caution.

### Measures

All constructs were measured using well-established scales adapted to the context of AI-generated art and creative design. The detail of the complete survey instrument, including all measurement items used in the study, has been provided in Appendix A for clarity and transparency. Participants completed a paper-based questionnaire in which they responded to items related to perceived usefulness of AI-generated art tools, artistic perception, cognitive engagement, aesthetic judgment, and self-reported creative design performance using a seven-point Likert scale (1 = strongly disagree, 7 = strongly agree).

Perceived usefulness of AI-generated art tools was measured by adapting the Perceived Usefulness subscale from the Technology Acceptance Model (Davis, 1989), which reliably captures how digital tools enhance task performance. Four items were reworded to fit creative-design tasks. A sample item is: “Using AI-generated art tools improves the quality of my design work.”

Artistic perception was assessed using items from the Perceptual Component of the Aesthetic Experience Questionnaire (AEQ) (Wanzer et al., 2020), which measures heightened attention to artistic form and visual elements. A sample item is: “I pay close attention to the shapes, colors, and visual structure of AI-generated artworks.”

Cognitive engagement was measured with the Situational Cognitive Engagement Scale (Rotgans and Schmidt, 2011), which captures focused mental involvement in a task. Items were adapted for design activities. A sample item is: “I was mentally absorbed while working on the AI-assisted design task.”

Aesthetic judgment was measured using the Aesthetic Judgment Style Scale (Bahrami-Ehsan et al., 2015), which evaluates analytical and emotional depth in evaluating artworks. A sample item is: “I consider both meaning and emotional impact when evaluating AI-generated images.”

Self-reported creative design performance was measured using the well-known creativity scale developed by Zhou and George (2001), which assesses novelty and usefulness of creative output. Items were adapted to design performance. A sample item is: “I generate original and practical visual ideas for design tasks.”

### Control

To reduce the potential influence of individual differences in AI-related experience, several control variables were included in the analysis, including familiarity with AI-generated art tools, frequency of AI tool usage, and prior AI-related training experience. These variables were controlled because prior exposure to AI-assisted creative technologies may influence perceptual, cognitive, and evaluative responses during creative design activities.

The control variables were incorporated into the structural model during hypothesis testing. The inclusion of these controls did not materially alter the significance or direction of the hypothesized relationships, indicating that the proposed associations remained stable after accounting for prior AI-related exposure and experience.

### Analytical strategy

Data were analyzed using SmartPLS 4.0. PLS-SEM was considered appropriate because the study aimed to simultaneously examine multiple mediating relationships and assess the model’s predictive and explanatory capability within an emerging research context involving AI-generated art and creative cognition (Hair et al., 2019). In addition, PLS-SEM is considered suitable for models that incorporate multiple latent constructs and indirect pathways while placing emphasis on variance explanation and prediction-oriented analysis rather than strict model fit reproduction associated with covariance-based SEM (Kock, 2018). The structural model was evaluated using SmartPLS 4.0 through a bootstrapping procedure with 5,000 bootstrap resamples, bias-corrected confidence intervals, and two-tailed significance testing at the 95% confidence level. In addition to reliability and validity assessment, model evaluation included SRMR, NFI, RMS_theta, *R*^2^, *f*^2^, *Q*^2^, and HTMT values based on recommended threshold criteria reported in prior PLS-SEM literature (Hair et al., 2019). Furthermore, PLSpredict analysis demonstrated positive *Q*^2^ predict values across the endogenous constructs, supporting the model’s predictive relevance. Furthermore, RMSE and MAE comparisons between the PLS-SEM predictions and benchmark linear model predictions indicated acceptable predictive performance across the endogenous constructs, providing additional support for the model’s out-of-sample predictive capability.

## Results

The measurement model was assessed through outer loadings, internal consistency reliability, and convergent validity (Table 1). All item loadings exceeded the recommended threshold of 0.70, ranging from 0.723 to 0.903, indicating satisfactory indicator reliability. Cronbach’s alpha values for all constructs fell between 0.842 and 0.892, while composite reliability values ranged from 0.892 to 0.927, demonstrating high internal consistency. The AVE values exceeded the recommended minimum of 0.50, confirming the presence of convergent validity. Together, these results confirm that the measurement model is reliable and capable of capturing the intended constructs.

**Table 1:** Measurement model assessment.

Construct	Item	Loading	CA	CR	AVE
Perceived usefulness of AI-generated art tools	AGA1	0.812	0.842	0.892	0.674
	AGA2	0.837			
	AGA3	0.846			
	AGA4	0.789			
Artistic perception	AP1	0.803	0.861	0.907	0.712
	AP2	0.857			
	AP3	0.874			
	AP4	0.723			
Cognitive engagement	CE1	0.821	0.847	0.900	0.695
	CE2	0.854			
	CE3	0.792			
	CE4	0.833			
Aesthetic judgment	AJ1	0.804	0.876	0.917	0.736
	AJ2	0.893			
	AJ3	0.854			
Self-reported creative design performance	CD1	0.846	0.892	0.927	0.759
	CD2	0.903			
	CD3	0.874			

Multicollinearity was examined using variance inflation factor (VIF) scores (Table 2). All VIF values fell well below the conservative threshold of 3.00, ranging between 1.833 and 2.903. This indicates that multicollinearity was not problematic in the dataset and that each indicator uniquely contributed to its respective latent variable without redundant overlap.

**Table 2:** Multicollinearity assessment.

Construct	Item	VIF
Perceived usefulness of AI-generated art tools	PU1	2.013
	PU2	2.187
	PU3	2.224
	PU4	1.964
Artistic perception	AP1	2.116
	AP2	2.384
	AP3	2.457
	AP4	1.833
Cognitive engagement	CE1	2.041
	CE2	2.267
	CE3	1.924
	CE4	2.153
Aesthetic judgment	AJ1	2.228
	AJ2	2.671
	AJ3	2.416
Self-reported creative design performance	CD1	2.548
	CD2	2.903
	CD3	2.671

To further assess the potential risk of common method bias, both procedural and statistical remedies were applied. Procedurally, respondents were assured of anonymity and confidentiality to reduce evaluation apprehension and social desirability bias. Statistically, Harman’s single-factor test was conducted, and the first unrotated factor accounted for 34.21% of the total variance, which remained below the recommended threshold of 50%, indicating that common method bias was unlikely to be a serious concern.

Discriminant validity was examined using both the Fornell–Larcker criterion (Table 3a) and the HTMT ratio (Table 3b). The square roots of the AVE values were consistently greater than the corresponding inter-construct correlations, providing evidence of discriminant validity. In addition, all HTMT ratios were below 0.85, further confirming that the constructs are empirically distinct from one another. These results together demonstrate that each construct possesses adequate discriminant validity and measures a unique conceptual domain within the model.

**Table 3a:** Discriminant validity—Fornell-Larcker.

Construct	Perceived usefulness of AI-generated art tools	Artistic perception	Cognitive engagement	Aesthetic judgment	Self-reported creative design performance
Perceived usefulness of AI-generated art tools	0.821				
Artistic perception	0.516	0.844			
Cognitive engagement	0.544	0.622	0.834		
Aesthetic judgment	0.491	0.598	0.606	0.858	
Self-reported creative design performance	0.563	0.614	0.641	0.629	0.871

**Table 3b:** Discriminant validity—HTMT ratio.

Construct pair	HTMT
Perceived usefulness of AI-generated art tools → artistic perception	0.612
Perceived usefulness of AI-generated art tools → cognitive engagement	0.648
Perceived usefulness of AI-generated art tools → aesthetic judgment	0.573
Perceived usefulness of AI-generated art tools → creative design	0.682
Artistic perception → cognitive engagement	0.731
Artistic perception → aesthetic judgment	0.694
Artistic perception → self-reported creative design performance	0.722
Cognitive engagement → aesthetic judgment	0.705
Cognitive engagement → self-reported creative design performance	0.758
Aesthetic judgment → self-reported creative design performance	0.741

Model fit indicators were used to assess the overall adequacy of the PLS-SEM model (Table 4). The SRMR value of 0.047 fell comfortably below the threshold of 0.08, indicating good model fit. The NFI value of 0.914 further supported the acceptability of the model, while the RMS_theta value of 0.112 remained under the recommended cutoff, suggesting satisfactory indicator quality. Additional fit indicators such as d_ULS and d_G were low, reinforcing the appropriateness of the structural model for capturing the relationships among constructs.

**Table 4:** Model fit.

Fit index	Value	Recommended threshold	Interpretation
SRMR	0.047	< 0.080	Good model fit
NFI	0.914	> 0.900	Acceptable fit
RMS_theta	0.112	< 0.120	Acceptable indicator quality
Chi-square	1,287.436	—	Reported for completeness
d_ULS	0.982	Lower is better	Good
d_G	0.731	Lower is better	Good

The structural model demonstrated meaningful explanatory power for all endogenous constructs (Table 5a). The *R*^2^ values indicated that 26.6 percent of the variance in artistic perception, 29.6 percent of the variance in cognitive engagement, and 24.1 percent of the variance in aesthetic judgment were explained by perceived usefulness of AI-generated art tools. More notably, the model accounted for 53.2 percent of the variance in self-reported creative design performance, which represents a substantial level of explanatory strength in behavioral research. These *R*^2^ results indicate that the proposed model provides a strong theoretical foundation for understanding associations between AI-generated art tools and cognitive and aesthetic processes that shape self-reported creative design outcomes.

**Table 5a:** Structural model summary.

Endogenous construct	*R* ^2^	Interpretation
Artistic perception	0.266	Moderate
Cognitive engagement	0.296	Moderate
Aesthetic judgment	0.241	Moderate
Self-reported creative design performance	0.532	Substantial

Effect size values revealed that perceived usefulness of AI-generated art tools demonstrated strong positive associations with these mediators, indicating its strong capacity to shape artistic perception, cognitive engagement, and aesthetic judgment (Table 5b). The mediators themselves exerted medium effect sizes on creative design performance, suggesting that each contributed meaningfully to the outcome variable. The direct effect of perceived usefulness of AI-generated art tools on self-reported creative design performance was small to medium, consistent with the expectation that much of the influence is transmitted through the mediating mechanisms.

**Table 5b:** Effect size.

Relationship	*f* ^2^	Effect
Perceived usefulness of AI-generated art tools → artistic perception	0.362	Large
Perceived usefulness of AI-generated art tools → cognitive engagement	0.421	Large
Perceived usefulness of AI-generated art tools → aesthetic judgment	0.317	Medium–large
Artistic perception → self-reported creative design performance	0.144	Medium
Cognitive engagement → self-reported creative design performance	0.203	Medium
Aesthetic judgment → self-reported creative design performance	0.176	Medium
Perceived usefulness of AI-generated art tools → self-reported creative design performance (direct)	0.118	Small–medium

Predictive relevance was examined using Stone-Geisser’s *Q*^2^ values (Table 5c). All endogenous constructs demonstrated *Q*^2^ values above zero, indicating predictive relevance for the model. Artistic perception, cognitive engagement, and aesthetic judgment exhibited small to medium levels of predictive relevance, while self-reported creative design performance demonstrated strong predictive capability with a *Q*^2^ value of 0.347. These results collectively indicate that the model possesses both explanatory and predictive power.

**Table 5c:** Predictive relevance.

Construct	*Q* ^2^	Interpretation
Artistic perception	0.168	Medium predictive relevance
Cognitive engagement	0.192	Medium predictive relevance
Aesthetic judgment	0.151	Small–medium relevance
Self-reported creative design performance	0.347	Strong predictive relevance

In addition to Stone-Geisser’s *Q*^2^, PLSpredict analysis further supported the predictive capability of the proposed model, as all endogenous constructs demonstrated positive *Q*^2^ predict values above zero. These findings indicate that the model possesses acceptable out-of-sample predictive relevance for the examined relationships.

The structural path coefficients, shown in Table 6, indicated that perceived usefulness of AI-generated art tools was positively associated with all three mediators: artistic perception (*β* = 0.514, *p* < 0.001), cognitive engagement (*β* = 0.544, *p* < 0.001), and aesthetic judgment (*β* = 0.491, *p* < 0.001). Each mediator also significantly predicted self-reported creative design performance, with cognitive engagement emerging as the strongest mediator (*β* = 0.297, *p* < 0.001), followed by aesthetic judgment (*β* = 0.263, *p* < 0.001) and artistic perception (*β* = 0.214, *p* = 0.001). The direct effect of perceived usefulness of AI-generated art tools on self-reported creative design performance remained significant (*β* = 0.181, *p* = 0.006), indicating partial mediation. This pattern suggests that perceived usefulness of AI-generated art tools is associated with self-reported creative design performance both directly and indirectly through cognitive and perceptual pathways.

**Table 6:** Path coefficients, *t*-values, and *p*-values.

Path	*β*	*t*-value	*p*-value
Perceived usefulness of AI-generated art tools → artistic perception	0.514*	12.442	< 0.001
Perceived usefulness of AI-generated art tools → cognitive engagement	0.544*	13.118	< 0.001
Perceived usefulness of AI-generated art tools → aesthetic judgment	0.491*	11.027	< 0.001
Artistic perception → self-reported creative design performance	0.214	3.228	0.001
Cognitive engagement → self-reported creative design performance	0.297*	4.911	< 0.001
Aesthetic judgment → self-reported creative design performance	0.263*	4.382	< 0.001
Perceived usefulness of AI-generated art tools → self-reported creative design performance (direct)	0.181	2.744	0.006

*p < 0.01.

In addition to path coefficients and significance values, all direct and indirect effects were examined using 95% bias-corrected confidence intervals, and none of the intervals included zero, further supporting the statistical significance and robustness of the hypothesized relationships.

The mediation analysis revealed that all three mediators carried significant indirect effects (Table 7). The indirect association through artistic perception was significant (*β* = 0.110, *p* = 0.003), as were the effects through cognitive engagement (*β* = 0.162, *p* < 0.001) and aesthetic judgment (*β* = 0.129, *p* < 0.001). The total indirect effect of 0.401 was substantial and exceeded the magnitude of the direct effect, highlighting the central role of cognitive and aesthetic mechanisms in explaining the association between perceived usefulness of AI-generated art tools and self-reported creative design outcomes.

**Table 7:** Specific and total indirect effects.

Indirect path	Specific indirect effect (*β*)	*t*-value	*p*-value
Perceived usefulness of AI-generated art tools → artistic perception → self-reported creative design performance	0.110	3.012	0.003
Perceived usefulness of AI-generated art tools → cognitive engagement → self-reported creative design performance	0.162	4.221	< 0.001
Perceived usefulness of AI-generated art tools → aesthetic judgment → self-reported creative design performance	0.129	3.884	< 0.001
Total indirect effect	0.401	8.915	< 0.001

To further examine the appropriateness of the proposed model structure, the potential for a sequential relationship among the mediators was also considered conceptually. However, the parallel mediation framework was retained because artistic perception, cognitive engagement, and aesthetic judgment were theorized as distinct yet complementary psychological responses that may occur simultaneously during interaction with AI-generated artistic stimuli. This approach was considered more appropriate for capturing the unique explanatory contribution of each mediator within the present study.

To further evaluate the appropriateness of the proposed mediation structure, an alternative sequential mediation specification was also examined conceptually, in which artistic perception, cognitive engagement, and aesthetic judgment were treated as sequentially linked mechanisms. However, the parallel mediation framework demonstrated stronger explanatory clarity and theoretical consistency with prior creativity research suggesting that perceptual, cognitive, and evaluative responses may occur simultaneously during interaction with complex visual stimuli (Amabile and Pratt, 2016; Pelowski et al., 2017). In addition, the parallel specification provided clearer interpretation of the unique contribution of each mediator to self-reported creative design performance. Therefore, the proposed parallel mediation model was retained as the more theoretically and analytically appropriate framework for the present study.

## Discussion

### Theoretical implications

The results collectively suggest that perceived usefulness of AI-generated art tools is meaningfully associated with self-reported creative design performance rather than merely a technical add-on, thus offering substantial theoretical implications. The significant direct association between perceived usefulness of AI-generated art tools and self-reported creative design performance aligns with prior work showing that diverse and unconventional visual stimuli can broaden ideational search and support more original outcomes (Ashrafganjouei and Nadimi, 2021; Gerver et al., 2023). Studies in digital and AI-assisted creativity have reported that generative tools help learners explore alternative directions, break habitual patterns, and access a wider range of stylistic options (Grassini and Koivisto, 2025; Hou and Wang, 2025). The present findings converge with this stream by demonstrating that, even when controlling for internal psychological mechanisms, greater perceived usefulness of AI-generated art tools is associated with higher self-reported creative design performance. At the same time, the modest size of the direct path suggests that AI-generated imagery does not automatically translate into better designs; instead, its influence is partly contingent on how individuals process and utilize these visual inputs.

Patterns observed for artistic perception provide more fine-grained support for theoretical claims that perceptual sensitivity is a core ingredient of visual creativity. The positive link between perceived usefulness of AI-generated art tools and artistic perception is consistent with evidence that visually complex, unfamiliar, or stylistically disruptive stimuli sharpen perceptual attention and encourage more detailed inspection of formal features (Rueda-Arango et al., 2024). The finding that artistic perception, in turn, is associated with self-reported creative design performance resonates with prior work highlighting that designers who exhibit higher aesthetic sensitivity tend to produce more nuanced and coherent visual outcomes (Van Hees et al., 2025). By showing that artistic perception partially mediates the relationship between perceived usefulness of AI-generated art tools and self-reported creative design performance, this study extends previous research in two ways. First, it empirically confirms that AI-generated imagery contributes to creativity not only by expanding the volume of visual options but also by deepening the way learners perceive and decode artistic elements. Second, it positions perceptual refinement as a concrete psychological pathway through which generative tools become pedagogically meaningful in design education.

Cognitive engagement emerged as the strongest mediator, which offers an important extension to current discussions around AI and creativity. Prior studies in design cognition and learning have emphasized that deep mental involvement—characterized by focused attention, sustained effort, and active elaboration—supports higher-quality creative outcomes (Chandrasekera et al., 2025; Han et al., 2024). The positive association between perceived usefulness of AI-generated art tools and cognitive engagement observed here is in line with empirical reports that generative tools stimulate curiosity and analytical thinking by presenting surprising and sometimes challenging imagery (Yu, 2025). The fact that cognitive engagement shows the largest indirect effect suggests that the primary value of AI-generated art may lie in its ability to pull learners into a deeper cognitive relationship with their design tasks. Rather than replacing thinking, AI-generated stimuli appear to be associated with deeper cognitive involvement. This finding counters more pessimistic narratives that AI might reduce cognitive effort by automating creative work; instead, the results indicate that, under appropriate conditions, generative outputs may correspond with higher rather than lower cognitive engagement.

Aesthetic judgment also played a significant mediating role, reinforcing arguments that evaluative processes are central to creative practice. Prior research has stressed that the ability to critically assess visual coherence, expressive impact, and stylistic fit distinguishes more accomplished creators from less experienced ones (Han et al., 2024; Savaş et al., 2021). The present study shows that perceived usefulness of AI-generated art tools is positively associated with aesthetic judgment, suggesting that frequent interaction with algorithmically produced imagery encourages more deliberate reflection on what counts as “good” or meaningful design. This is consistent with recent work indicating that exposure to a wide range of AI-generated styles prompts users to compare, contrast, and evaluate visual alternatives more actively (Brielmann et al., 2024). The mediation results extend this literature by demonstrating that aesthetic judgment is not merely a by-product of creative activity but a functional mechanism through which AI-based stimuli influence final design quality. In other words, generative art appears to be associated with more deliberate evaluative reflection through which designers filter and refine ideas.

The overall pattern of partial, parallel mediation—where artistic perception, cognitive engagement, and aesthetic judgment all carry significant indirect effects while the direct path remains significant—suggests a multifaceted influence of AI-generated art. This configuration indicates that AI-based stimuli contribute to creative design in at least two ways: directly, by enriching the pool of available visual possibilities, and indirectly, by shaping how learners perceive, think about, and evaluate those possibilities. Compared with prior work that tends to emphasize a single mechanism (for example, inspiration or efficiency), the current study offers a more integrated view by modeling multiple psychological pathways simultaneously. This adds nuance to the emerging AI-creativity literature, showing that generative tools are most effective when they are embedded in processes that foster perceptual sharpening, cognitive immersion, and evaluative reflection.

Findings should also be interpreted in light of the Chinese higher education context in which the data were collected. Design, media, and digital-art programs in China are rapidly adopting AI tools, often encouraging students to experiment with generative imagery alongside traditional methods. This setting may intensify both the novelty and ubiquity of AI-generated art, potentially strengthening the observed associations with perception, engagement, and judgment. While this context supports the argument that AI can be productively integrated into design curricula, it also raises questions about boundary conditions: for example, whether similar patterns would emerge in environments where AI use is more restricted, or in professional studios where commercial constraints shape how generative outputs are used. These considerations open avenues for future research but do not diminish the central conclusion that AI-generated art, when meaningfully incorporated into learning and design practice, can enhance creative design performance through interconnected perceptual, cognitive, and aesthetic mechanisms.

### Practical implications

The findings offer several practical implications for educators, curriculum designers, and creative-industry professionals seeking to integrate AI-generated art into design practice. The strong role of cognitive engagement highlights that AI tools are most beneficial when they serve as stimuli that prompt deeper thinking rather than as shortcuts for producing final outputs. In educational settings, instructors can incorporate AI-generated imagery as part of structured exploration activities, such as guided critique sessions, comparative analysis exercises, or iterative redesign tasks. These activities can help students approach AI outputs analytically, encouraging them to question visual complexities, interpret stylistic choices, and develop stronger cognitive involvement in the design process. Such practices support the development of self-directed creative reasoning, rather than passive consumption of AI-generated material.

The meaningful contribution of artistic perception suggests that AI-generated art should be intentionally used to enhance students’ sensitivity to visual cues. Educators and design mentors can curate AI-generated references that highlight specific artistic qualities—such as color relationships, compositional balance, or stylistic contrast—to train perceptual attention. Assignments that require students to dissect AI-generated images, identify embedded aesthetic structures, or replicate stylistic elements in their own work may help cultivate perceptual refinement. These strategies are particularly relevant in introductory design courses, where building foundational visual awareness is critical to progress in later stages of creative training.

The observed influence of aesthetic judgment also underscores the importance of strengthening students’ evaluative skills. Institutions can incorporate AI-generated art into critique-based learning environments where students compare multiple AI outputs, justify their aesthetic preferences, and articulate why certain visual directions are more compelling or effective. Professional design teams can use generative tools to expand the range of early-stage concepts, followed by collaborative evaluation sessions where designers critique AI-generated options. This reflective process not only improves aesthetic reasoning but also ensures that AI is used to support, rather than dilute, design quality.

The partial mediation results suggest that perceived usefulness of AI-generated art tools is associated with self-reported creative design performance both directly and through internal psychological pathways. This suggests that design schools and creative organizations should adopt hybrid workflows where AI serves as a source of inspiration rather than a complete automation tool. Encouraging designers to iteratively alternate between AI-generated stimuli and manual refinement can maximize creative benefits while maintaining human-centered decision-making. Such workflows reduce the risk of over-reliance on AI defaults and promote deeper levels of cognitive, perceptual, and evaluative engagement.

Finally, the strong overall explanatory power of the model (R^2^ = 0.532) indicates that integrating AI-generated art into design education and practice may meaningfully enhance creative outcomes when used thoughtfully. Institutions should therefore consider formal training programs that equip learners and practitioners with the skills to critically analyze, interpret, and utilize generative imagery. As AI continues to reshape visual culture, cultivating these psychological capabilities will be essential for ensuring that creative workers remain adaptive, reflective, and empowered in an AI-mediated creative landscape.

### Limitations

Although this study provides meaningful insights into how perceived usefulness of AI-generated art tools is associated with self-reported creative design performance through perceptual, cognitive, and aesthetic mechanisms, several limitations should be acknowledged when interpreting the findings. The sample was drawn exclusively from Chinese university students in design-related programs, which may limit the generalizability of the results to other cultural, educational, or professional contexts where AI adoption, visual literacy training, and design workflows differ. Self-reported measures may also introduce common method bias, although the multi-construct model structure reduces this risk. In addition, the study focused on short-term psychological processes activated during interaction with AI-generated imagery; future research would benefit from longitudinal or experimental designs to examine how sustained exposure influences skill development, creative habits, or aesthetic preferences over time. The model also did not incorporate potential boundary conditions—such as prior artistic training, digital proficiency, or attitudes toward AI—that may shape the strength of these relationships. Last but not the least, because the study relied on a cross-sectional self-report design, the findings should be interpreted as associative rather than causal, and the mediation pathways should not be understood as evidence of true temporal mediation. Addressing these limitations in future studies would strengthen the theoretical scope and practical relevance of this emerging research area.

## Conclusion

This study demonstrates that perceived usefulness of AI-generated art tools is positively associated with self-reported creative design performance through interconnected pathways involving artistic perception, cognitive engagement, and aesthetic judgment. Rather than functioning merely as an efficiency tool, AI-generated imagery may be associated with deeper perceptual attention, sustained cognitive involvement, and stronger evaluative reasoning—processes that are associated with higher self-reported creative design outcomes. The significant direct and indirect effects highlight that AI serves both as a source of inspiration and as a catalyst for internal psychological mechanisms that support creative work. These findings advance current understanding of AI-assisted creativity by providing a more integrated view of how generative tools relate to the human dimensions of design practice. As AI continues to reshape visual industries, recognizing and leveraging these mechanisms will be essential for educators, practitioners, and researchers seeking to cultivate creativity in increasingly AI-mediated design environments.

## Data Availability

The raw data supporting the conclusions of this article will be made available by the authors, without undue reservation.

## Appendix A: measurement items

### Perceived usefulness of AI-generated art tools

PU1: Using AI-generated art tools improves the quality of my design work.

PU2: AI-generated art tools help me develop creative design ideas more effectively.

PU3: AI-generated art tools enhance my efficiency during design tasks.

PU4: Overall, AI-generated art tools are useful in my creative design activities.

### Artistic perception

AP1: I pay close attention to the shapes, colors, and visual structure of AI-generated artworks.

AP2: I notice subtle artistic details in AI-generated images.

AP3: AI-generated artworks increase my sensitivity toward artistic elements and composition.

AP4: I carefully observe stylistic variations in AI-generated visual content.

### Cognitive engagement

CE1: I was mentally absorbed while working on AI-assisted design activities.

CE2: I concentrated deeply when interacting with AI-generated artistic content.

CE3: AI-generated visual materials encouraged me to think critically about design ideas.

CE4: I invested considerable mental effort while engaging with AI-generated imagery.

### Aesthetic judgment

AJ1: I consider both meaning and emotional impact when evaluating AI-generated images.

AJ2: I carefully judge the artistic quality of AI-generated visual outputs.

AJ3: AI-generated artworks encourage me to reflect on aesthetic balance and harmony.

### Self-reported creative design performance

CD1: I generate original and practical visual ideas for design tasks.

CD2: I often develop creative solutions during design-related activities.

CD3: I produce visually innovative outcomes in my creative work.
